# Impact of the COVID-19 Pandemic on Musculoskeletal Disorder-Related Absenteeism Among Pediatric Healthcare Workers

**DOI:** 10.3390/healthcare13101116

**Published:** 2025-05-11

**Authors:** Maria Valentina Popa, Irina Luciana Gurzu, Claudia Mariana Handra, Bogdan Gurzu, Alina Pleșea Condratovici, Mădălina Duceac (Covrig), Eva Maria Elkan, Dana Elena Mîndru, Vlad Andrei Dabija, Letiția Doina Duceac

**Affiliations:** 1 Doctoral School of Biomedical Sciences, “Dunărea de Jos” University of Galați, 47 Domnească Street, 800008 Galați, Romania; maria_valentina_popa@yahoo.com (M.V.P.); madalinaduceac@yahoo.ro (M.D.); 2 Department of Preventive Medicine and Interdisciplinarity, Discipline of Occupational Health, “Grigore T. Popa” University of Medicine and Pharmacy, 700115 Iasi, Romania; 3 Occupational Medicine Department, “Carol Davila” University of Medicine and Pharmacy, 050474 Bucharest, Romania; 4 Department of Morfofunctional Sciences, Faculty of Medicine, “Grigore T. Popa” University of Medicine and Pharmacy, 700115 Iasi, Romania; bgurzu@yahoo.com; 5 Faculty of Medicine and Pharmacy, “Dunărea de Jos” University of Galați, 47 Domnească Street, 800008 Galați, Romania; alina.plesea@ugal.ro (A.P.C.); cojocarumariaeva@yahoo.com (E.M.E.); letimedr@yahoo.com (L.D.D.); 6 Department of Pediatrics, Faculty of Medicine, “Grigore T. Popa” University of Medicine and Pharmacy, 700115 Iasi, Romania; mindru.dana@umfiasi.ro; 7 Radiology Department, “St. Spiridon” County Emergency Clinical Hospital, “Grigore T. Popa” University of Medicine and Pharmacy, 700115 Iasi, Romania; vlad-andrei.dabija@email.umfiasi.ro

**Keywords:** musculoskeletal disorders, absenteeism, pediatric healthcare worker, occupational health, COVID-19, rehabilitation, statistical modeling

## Abstract

**Background and Objectives:** Musculoskeletal disorders (MSDs) are a leading cause of absenteeism among healthcare workers (HCWs), impacting healthcare delivery. Pediatric HCWs face specific physical demands such as lifting and awkward postures. While absenteeism rose during the COVID-19 pandemic, its effects on pediatric MSD-related leave remain unclear. This study examined MSD-related absenteeism trends among pediatric HCWs in a Romanian hospital across the pre-pandemic (2017–2019), pandemic (2020–2021), and post-pandemic (2022–2023) periods. **Materials and Methods:** We conducted a retrospective observational study using records from the hospital’s occupational health database. We included all HCWs who took MSD-related leave during 2017–2023. Diagnoses included arthropathies, dorsopathies, other osteoarticular/connective tissue disorders, and acute trauma or fractures. We used chi-square tests, ANOVA, and regression models to identify trends and predictors. **Results:** A total of 3388 cases were analyzed. Post-pandemic absenteeism increased significantly (40.1%), especially among women (86.8%), nurses (46.7%), and workers aged ≥46 (62.7%). A seasonal shift was observed, with spring peaks (March 9.7% and May 9.9%) replacing the pre-pandemic autumn peaks (October 11.9% and November 12.8%). The regression models identified age, occupation, and diagnosis type as significant predictors of leave duration. **Conclusions:** MSD-related absenteeism rose post-pandemic and showed altered seasonal patterns. Occupational and demographic predictors identified through a multivariate analysis highlight the need for anticipatory, evidence-based strategies to support pediatric HCWs, enhance workforce resilience, and sustain healthcare performance.

## 1. Introduction

Musculoskeletal disorders (MSDs) are a common cause of work-related absenteeism among healthcare professionals (HCWs), significantly impacting both individual well-being and the overall efficiency of healthcare systems [1,2,3]. These conditions frequently affect the spine, neck, shoulders, and lower back and are often associated with manual patient handling, awkward postures, and extended shifts [4,5]. In pediatric healthcare settings, such risks are exacerbated by the physical demands of caring for children, who require frequent lifting, repositioning, and handling, often involving unpredictable movements [6,7].

Although pediatric HCWs are particularly vulnerable, the literature lacks detailed data on MSD-related absenteeism in this group, especially regarding the COVID-19 pandemic. Most studies to date have focused on the psychological burden of the pandemic, such as burnout and stress [8,9,10], with fewer examining physical health outcomes like MSDs or how the pandemic may have influenced absenteeism patterns by gender, age, occupation, or seasonality. No Romania-specific data exist on absenteeism due to MSDs among HCWs. Still, international studies highlight MSDs as a major global cause of absenteeism, especially among older and physically active HCWs [3,5,11]. Risk factors include biomechanical strain, psychosocial stress, chronic pain, and environmental discomforts [12,13,14,15,16,17]. Physical activity helps reduce absenteeism, though workplace interventions show mixed results [18,19,20].

Emerging research indicates that the pandemic has been associated with a rise in the prevalence and severity of MSDs [21,22,23]. Several mechanisms contribute to this trend: disruptions in access to healthcare services have led to delays in managing chronic musculoskeletal conditions [24,25]; increased workloads and stress among frontline workers have exacerbated physical strain [26,27]; and changes in work ergonomics due to infection control protocols have placed new demands on HCWs. Additionally, musculoskeletal symptoms are now recognized as components of post-COVID syndrome, such as joint pain, muscle fatigue, and generalized discomfort [28], disproportionately affecting healthcare workers [17], computer users [29], and COVID-19 survivors [30]. Thus, the pandemic introduced new occupational risks for MSDs and intensified the existing ones. Therefore, we hypothesize that the COVID-19 pandemic significantly influenced MSD-related absenteeism among pediatric HCWs, with variations observable across demographic characteristics, occupation, seasonality, and diagnostic categories.

## 2. Materials and Methods

### 2.1. Study Design

This retrospective observational study was conducted at the regional pediatric hospital in Iasi, located in the northeast region of Romania. The hospital employs approximately 1300 HCWs, of which about 86% are female and more than half (61%) are aged 46 or older, reflecting the general age and gender distribution of Romanian pediatric healthcare staff [31].

A total of 3388 sick leaves among pediatric HCWs with musculoskeletal disorders (MSDs) were analyzed (Table 1).

To analyze age-related patterns in absenteeism due to MSDs, the HCWs were divided into two groups based on the median age of the study population, which is 46 years. This data-driven categorization (<46 years vs. ≥46 years) allowed for balanced subgroup comparisons and aligns with standard epidemiological practices where no universally accepted age threshold exists for defining “younger” and “older” workers in the context of occupational MSD risk. Similar approaches have been used in published studies, such as Silva-Junior et al. (2023), who identified reduced return-to-work rates in healthcare workers aged ≥40 [32], and Zahrawi et al. (2024), who reported higher MSD prevalence with increasing age [33]. Furthermore, Zerbo Šporin et al. (2025) observed that older HCWs had longer durations of MSD-related sick leave without a fixed age cut-off [17]. Given this variability in the literature, using the sample median ensures consistency, transparency, and methodological rigor in an age-based analysis.

This study aims to enhance the understanding of changes in MSD-related absenteeism patterns in pediatric settings during and after the COVID-19 pandemic. Utilizing a retrospective analysis of medical leave records from a regional hospital in Romania, we examine the trends across three distinct periods: pre-pandemic (2017–2019), pandemic (2020–2021), and post-pandemic (2022–2023).

The study objectives are to (i) determine the distribution of MSD cases by diagnostic category (arthropathies, dorsopathies, other osteoarticular diseases, and trauma/fractures); (ii) identify key risk factors for absenteeism, including gender, age, and occupation; (iii) analyze seasonal variations in MSD-related absenteeism; and (iv) develop predictive models using statistical methods.

The hypotheses were investigated in a local/regional context based on existing data in the literature on absenteeism due to MSDs among health professionals in general [17,34]. The study of MSD-related sickness absence among workers in the pediatric hospital used three key variables. First, the total number of sick leaves taken by HCWs indicates workers’ health status and reflects the financial stability of the social security system. Second, the average number of calendar days spent on sick leave may reveal systemic problems in the healthcare system and their impact on public health. Third, the average number of missed working days is important for assessing the impact on hospital activity, as an HCW shortage may lead to lower quality of care and higher expenses. Additionally, we explored whether predictors of sickness absence could be modeled based on demographic and clinical characteristics.

This study aims to address the above aspects, contributing to more effective occupational health strategies and enhancing workforce resilience in pediatric healthcare.

### 2.2. Sample

This study included pediatric HCWs from various professional categories: physicians, nurses, nursing assistants, porters, caregivers, medical registrars, auxiliary personnel, and administrative staff.

#### 2.2.1. Inclusion Criteria

This study included hospital workers who received sick leave for MSDs during 2017–2023. The analyzed group consisted of HCWs from different professional categories, including physicians, nurses, nursing assistants, stretcher bearers, caregivers, medical registrars, auxiliary staff, and administrative staff. Only sickness absences for specific MSD diagnoses were included: arthropathies, dorsopathies, other osteoarticular and connective tissue disorders, and trauma or fractures. The study period was divided into three distinct phases: pre-pandemic (2017–2019), pandemic (2020–2021), and post-pandemic (2022–2023). HCWs with complete data on sickness duration (expressed in total calendar and working days), established diagnosis, gender, age, and job function were included. These criteria ensured a rigorous analysis of the factors influencing sickness absence among pediatric HCWs.

#### 2.2.2. Exclusion Criteria

Workers missing from work due to reasons other than MSDs, those not employed for the entire duration of any analyzed period, or those with temporary contracts were excluded from this study. Cases were also excluded from the analysis if essential information regarding sick absence (duration, diagnosis, age, gender, and occupation) was incomplete or unavailable. We did not consider sick leave when bone or muscle conditions were associated with other diagnoses that could have influenced the length of absence from work. HSWs on sick leave for pre-existing chronic conditions unrelated to work were also excluded.

These inclusion and exclusion criteria facilitated the selection of a homogeneous sample, ensuring an objective assessment of sickness absence due to MSDs and its impact on pediatric healthcare workers.

### 2.3. Data Collection

Data on MSD-related sick leave were automatically extracted from the hospital’s electronic medical record systems and occupational health databases. The dataset included information recorded as part of routine administrative and medical documentation without using questionnaires or direct medical consultations. Specialist clinicians (M.D.) conducted clinical examinations, laboratory tests, and imaging investigations required to support the diagnosis of musculoskeletal disorders (MSDs) according to national protocols. Diagnoses were documented using ICD-10 codes and verified by the hospital’s occupational health service. This study included hospital workers employed throughout the study period (2017–2023), while external contractors, temporary workers, and staff on administrative leave were excluded. The data collection included key variables such as diagnosis codes (ICD-classified musculoskeletal disorders), duration of sick leave (both calendar and working days), gender, age, and occupational role. All collected data were processed to remove errors, duplicates, and incomplete information. The diagnostic codes were thoroughly reviewed to ensure that only cases explicitly related to musculoskeletal disorders were included. Data that did not meet the inclusion criteria were excluded from the final dataset.

### 2.4. Data Analysis

The statistical analysis utilized descriptive and inferential methods to assess the distribution of sickness absence due to MSDs among hospital workers. The chi-squared test (χ^2^) was used to compare the distributions of categorical variables (such as gender, age group, occupation, and study period) in order to identify significant differences between groups. Differences in the means of the number of sick days (both calendar days and working days) were analyzed using a one-way ANOVA test. Logistic regression models estimated the probability of sick leave as a function of independent variables (gender, age, occupation, pandemic period, and season). Linear regression determined the influence of these variables on the number of working days of sick leave. Regression coefficients were expressed as odds ratios (ORs) with 95% confidence intervals (95% CI). The level of statistical significance was “*p* < 0.05”. The statistical analysis was performed using specialized software packages (IBM SPSS Statistics version 25.0), and the results were interpreted in the epidemiological and occupational context of pediatric healthcare.

### 2.5. Ethical Considerations

This study was conducted in accordance with the Declaration of Helsinki and with international ethical standards in medical research. All data were anonymized, and access to personal information was restricted. This study adhered to the principles of medical ethics and received approval from the hospital ethics committee (35983/13 December 2022).

### 2.6. Potential Biases and Data Limitations

While this study benefits from a large dataset and consistent diagnostic categories, the retrospective design that relies on anonymized administrative records presents inherent limitations. First, there may be inconsistencies in diagnostic coding or documentation errors within the electronic occupational health system. Although we manually verified the diagnosis codes to ensure only MSD-related absences were included, underreporting or misclassification cannot be entirely ruled out. Second, the dataset consists of multiple medical leaves per individual; however, repeated events were analyzed as independent observations. The introduction of clustering effects may impact the statistical significance of the regression model. Finally, the classification of the 2022 data as “post-pandemic” may overlap with lingering pandemic effects, slightly blurring the contrast between study periods.

## 3. Results

The 217 medical certificates diagnosing arthropathy were more common in the post-pandemic period (39.2%), particularly among women (92.1%), individuals aged over 46 years (81.5%), and those in the nursing profession (52.5%). The 2116 medical certificates diagnosing dorsopathy were more frequent in the post-pandemic period (39%), primarily among women (87.2%), individuals aged over 46 years (62.7%), and those with a registered nurse occupation (46.6%). The 147 medical certificates diagnosing other diseases of the musculoskeletal system and connective tissue were more frequent in the post-pandemic period (44.9%), especially among women (93.2%), individuals aged over 46 years (61.9%), and among nurses (54.4%). The 908 medical certificates diagnosing acute pathology and trauma/fractures were more frequent in the post-pandemic period (42.2%), particularly among women (83.4%), those aged over 46 years (58.1%), and nurses (44.4%) (Table 1).

The univariate ANOVA analysis demonstrated that most medical leaves were recorded in the post-pandemic period (40.1%; *p* = 0.001), among females (86.8%; *p* = 0.001), for individuals over 46 years of age (62.7%; *p* = 0.001), and among nurses (46.7%; *p* = 0.025) (Table 1).

**Figure 1:**
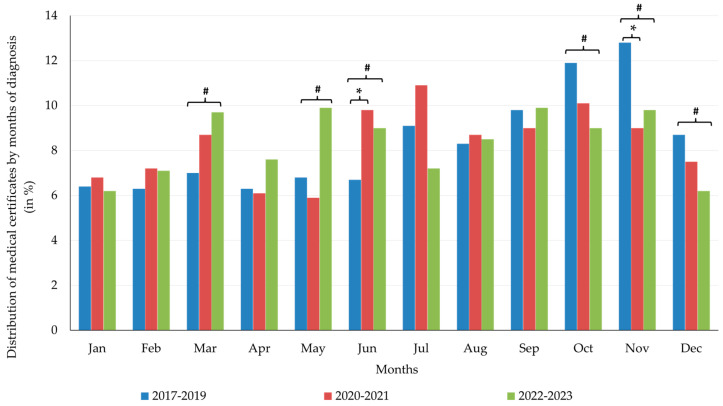
Medical certificates distributed by months of diagnosis for each of the pre-pandemic (2017–2019), pandemic (2020–2021), and post-pandemic (2022–2023) periods in the analyzed pediatric hospital. * *p* < 0.05 pre-pandemic vs. pandemic. # *p* < 0.05 post-pandemic vs. pre-pandemic.

Monthly distribution of certificates diagnosing MSDs (Figure 1):

- For the 1019 certificates from the pre-pandemic period (2017–2019), the most frequent months were March (7%), July (9.1%), and November (12.8%) (*p* = 0.01);
- For the 1009 certificates from the pandemic period (2020–2021), the most frequent months were March (8.7%), July (10.9%), and October (10.1%) (*p* = 0.073);
- For the 1360 certificates in the post-pandemic period (2022–2023), the most frequent months were March (9.7%), May (9.9%), and September (9.9%) (*p* = 0.001).

March and May recorded a notable rise in absenteeism during the post-pandemic period. Following a significant increase during the pandemic, June shows only a moderate rise in absenteeism, while the trend reverses in July. October, November, and December demonstrated significant decreases in the post-pandemic period compared to pre-pandemic levels. These data suggest that the seasonality of sickness absence shifted after the pandemic, with increased absenteeism from March to May, while the late autumn and winter months (November to December) exhibited lower rates than in the pre-pandemic years. January–February and August–September experience minor fluctuations.

**Figure 2:**
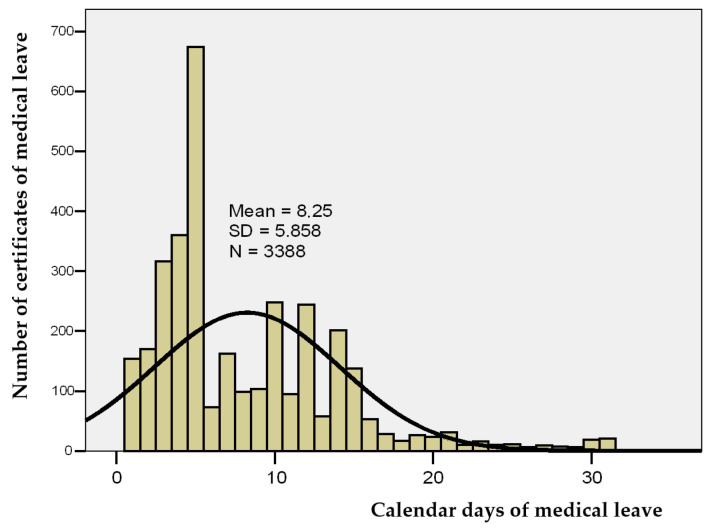
Histogram of calendar days of sick leave.

In Romania, calendar days of sickness absence refer to all consecutive days on which a hospital worker is temporarily unable to work, including working days, weekends, and public holidays. In the studied group, the number of calendar days of sick leave ranged from 1 to 31 days, with a group mean of 8.25 days ± 5.86, a median of 6 days, and a skewness test result of *p* = 1.335, indicating that the range of values was homogeneous, allowing for tests of statistical significance to be applied (Table 2 and Figure 2).

**Figure 3:**
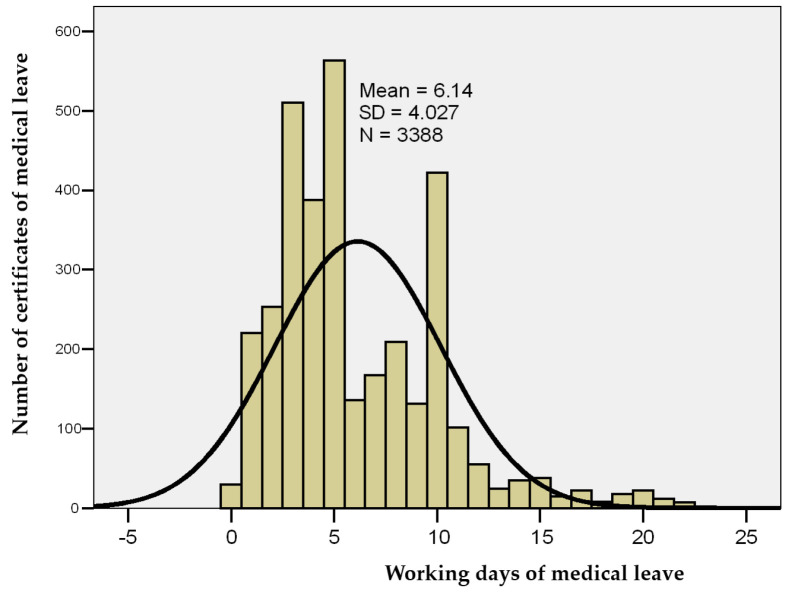
Histogram of working days of sick leave.

In Romania, working days of sick leave refer to the actual days that a hospital worker should have worked but is temporarily unable to do so due to paid sick leave. These days exclude weekends and public holidays unless they are considered working days according to the HCW’s employment contract. Within the studied group, the number of working days of sick leave ranged from none to 23 days, with a group mean of 6.14 days ± 4.03, a median of 5 days, and a skewness test result of *p* = 1.160, indicating that the series of values was homogeneous, thus allowing for the application of statistical significance tests (Table 3 and Figure 3).

According to the causes of absenteeism, the univariate analysis reveals that the lowest mean value of sick leave days is found in patients with dorsopathies (5.69 ± 3.43 days), while the highest is in patients with arthropathies (7.53 ± 4.41 days) (*p* = 0.001). Additionally, the lowest mean number of calendar days of sick leave was observed in patients with dorsopathies (7.38 ± 4.86 days), whereas the highest was seen in patients with arthropathies (10.14 ± 6.24 days) (*p* = 0.001) (Table 4).

Overall, the average duration of sick leave among the staff of Iași Children’s Hospital progressively decreased over the analyzed periods (2017–2019 vs. 2020–2021 vs. 2022–2023) (Table 5). A noticeable decrease in the average number of sick days during the pandemic and post-pandemic periods is observed for both males and females compared to the pre-pandemic period. For example, male staff experienced a decline from an average of 9.56 days (±7.19) before the pandemic to 7.41 days during the pandemic and 6.96 days after the pandemic. Female staff also demonstrated a similar trend (from 8.92 ± 6.15 days to 7.87 ± 5.66 days post-pandemic), although the absolute decrease for women was more modest. Staff age influences these trends: younger employees (<46 years) had relatively constant mean values of sickness absence (8.56 days before the pandemic vs. 7.62 days after the pandemic, showing small variation). In contrast, older healthcare workers (≥46 years) experienced a more pronounced reduction in sickness absence duration, from 9.42 ± 6.14 days before the pandemic to 7.83 ± 5.62 days after the pandemic. Thus, older workers, who initially took longer sick leave, reduced their sick leave to nearly the same level as their younger colleagues by the end of the study period. Different trends emerged depending on the diagnoses of the illnesses that led to sick leave. The most common musculoskeletal categories were arthropathies (degenerative joint diseases), dorsopathies (spinal disorders), other osteoarticular conditions, and acute trauma (including fractures). Among these, dorsopathies showed a consistent decrease in sick absence: from an average of ~7.95 days before the pandemic to 6.86 days after. Sick leave for acute trauma also saw a reduction in average duration over the same period (from ~10.72 days to 9.21 days). In contrast, absenteeism due to arthropathies remained relatively high and almost unchanged (around 10 days both before and after the pandemic), while absenteeism due to other osteo-articular conditions did not vary significantly (around 8.3 days on average). This suggests that certain chronic osteo-articular conditions maintained a constant level of absenteeism, whereas acute conditions or those that can improve with activity modification showed improvement during the pandemic period. There were also notable differences among staff categories (professions). Physicians experienced a significant decrease in the average duration of sick leave, from ~8.55 ± 7.05 days in 2017–2019 to just 5.20 ± 4.10 days after the pandemic. Caregivers with physically demanding jobs—the combined category of nurses/healthcare workers—also experienced a decrease in sick leave, from 10.20 ± 6.68 days (pre-pandemic) to 7.84 ± 5.53 days post-pandemic. Conversely, nurses (average staff) remained virtually at the same level throughout the period (~8.8 days before vs. 8.35 days after), suggesting stability in nurses’ absenteeism.

The category of administrative staff showed an atypical trend: the average duration of sick leave decreased significantly during the pandemic years (from 8.76 to 6.28 days) but then returned almost to baseline in 2022–2023 (8.73 days). Ancillary staff (e.g., cleaning and maintenance staff) and medical examiners/registrars exhibited a slight increase in average sick leave in the post-pandemic period compared to the pre-pandemic period (from lower values of approximately 6–7 days to about 8 days). Finally, the category “other highly educated professionals” (e.g., biologists and psychologists) consistently had the fewest days of sick leave, averaging around 5 days, with no significant variation between periods. The statistical analysis (one-way ANOVA test) confirms that many of the observed changes are statistically significant. For both male and female HCWs, the differences between the three periods (pre-, intra-, and post-pandemic) are significant (*p* = 0.001 for the downward trend for both genders). In other words, the average decrease in sick days for men and women during the pandemic and post-pandemic periods is not due to chance, indicating a real effect associated with the pandemic period. By age, the reduction in sick leave is statistically significant in the age group ≥46 years (*p* = 0.001), suggesting a genuine improvement in the health status or work availability of older workers during the pandemic years. For the age group <46 years, the variations did not reach the significance threshold (*p* = 0.069), indicating that the variation in sickness absence among younger staff was moderate and likely due to natural variability. Differences according to the medical condition causing the absence demonstrated that dorsopathies (back problems) experienced a significant decrease in absence duration between periods (*p* = 0.001), as did acute traumas (*p* = 0.023). These *p*-values below 0.05 confirm that the decrease in absence duration for back pain and trauma is statistically robust. In contrast, the ANOVA test for arthropathies and other osteo-articular conditions shows no significant differences between periods (*p* = 0.432 and *p* = 0.668, respectively), meaning that the small variations observed for these conditions may be random and not necessarily related to the pandemic context. An analysis by occupation also reveals important contrasts. The reduction in sickness absence among doctors is highly significant (*p* = 0.001), confirming the substantial decrease in sickness absence among doctors in 2020–2023 compared to the pre-pandemic period. There is also a statistically significant trend for non-physical hospital staff (nurses, orderlies, and medical assistants), where a real decrease in the duration of absence was observed (*p* = 0.001). Interestingly, the differences are also significant for administrative staff (*p* = 0.009), reflecting the large difference between the pandemic period (much reduced absence) and the other intervals. In contrast, no significant change was observed in sickness absence over time for nurses (*p* = 0.456), suggesting a relatively constant level of sickness absence. Similarly, medical consultants/registrars (*p* = 0.615), support staff (*p* = 0.549), and other categories with higher education did not exhibit statistically relevant differences between the analyzed periods, with variations too small to be considered significant. In interpreting these ANOVA results, very low *p*-values (<0.01) indicate a low probability that the observed differences are due to chance. Thus, we can assert with high confidence that, for example, the decrease in sickness absence among doctors or nurses is related to changes during the pandemic period. On the other hand, where *p* > 0.05, we cannot support the existence of a consistent trend (as is the case for nurses, younger staff, etc.), since the changes may be due to normal variability or other unstudied factors.

The predictive model for the cause of absence due to arthropathy, adjusted for sex, was built bottom-up, with the criterion for introducing a new variable being the LR (likelihood ratio) test. This was achieved by successively adding the study period, month of diagnosis, age group, number of calendar days, and number of working days of sick leave. The multivariate analysis suggests a significantly higher likelihood of arthropathy in women during the pandemic period (OR = 21.667; 95% CI: 6.810–68.936; *p* = 0.001); diagnosed in the months of May (OR = 14.804; 95% CI: 1.935–113.25; *p* = 0.009), November (OR = 11.541; 95% CI: 1.408–94.624; *p* = 0.023), July (OR = 10.381; 95% CI: 2.296–46.934; *p* = 0.002), and March (OR = 7.132; 95% CI: 1.993–25.514; *p* = 0.003); as well as among those aged 46 years and older (OR = 1.857; 95% CI: 1.039–3.526; *p* = 0.003) (Table 6).

The predictive model for the causes of absence due to dorsopathy, adjusted for sex, was built from the ground up. It employed the LR test as the criterion for introducing new variables, progressively adding the study period, month of diagnosis, age group, number of calendar days, and number of working days of sick leave. The multivariate analysis indicates a significantly higher likelihood of dorsopathy in women during the pandemic period (OR = 7.378; 95% CI: 5.858–9.292; *p* = 0.001), particularly diagnosed in February (OR = 13.539; 95% CI: 6.832–26.831; *p* = 0.001), July (OR = 9.271; 95% CI: 5.272–16.306; *p* = 0.001), March (OR = 8.129; 95% CI: 4.876–13.554; *p* = 0.001), November (OR = 7.416; 95% CI: 4.329–10.615; *p* = 0.001), and April (OR = 7.210; 95% CI: 4.170–12.465; *p* = 0.001), for individuals aged 46 years and older (OR = 1.302; 95% CI: 1.000–1.903; *p* = 0.006) (Table 7).

The model for predicting absenteeism due to diseases of the musculoskeletal system and connective tissue, adjusted for sex, was constructed in a bottom-up manner, using the LR test as the criterion for introducing new variables. The study period, month of diagnosis, age group, number of calendar days, and number of working days of sickness absence were added progressively. The multivariate analysis indicates a significantly higher likelihood in women during the pre-pandemic period (OR = 7.873; 95% CI: 5.358–69.893; *p* = 0.001), diagnosed in March (OR = 8.124; 95% CI: 1.010–65.317; *p* = 0.049) and September (OR = 8.836; 95% CI: 1.116–69.976; *p* = 0.039) (Table 8).

The model predicting the cause of absenteeism due to acute pathological trauma/fractures, adjusted by gender, was built bottom-up. The criterion for introducing a new variable was the LR test, gradually adding the study period, month of diagnosis, age group, number of calendar days, and the number of working days for sick leave. The multivariate analysis suggests a significantly higher likelihood among women in the pre-pandemic period (OR = 4.925; 95% CI: 3.665–6.616; *p* = 0.001), diagnosed in March (OR = 6.257; 95% CI: 3.000–13.051; *p* = 0.049) and November (OR = 6.252; 95% CI: 3.423–11.418; *p* = 0.039) (Table 9).

Linear regression models were developed for four diagnostic categories of musculoskeletal disorders (arthropathy, dorsopathy, other MSDs, and trauma/fractures) using the same set of predictors: pandemic period (Period), month of diagnosis (MonthDg), age, and number of calendar days of sick leave (DaysC). All models demonstrated strong predictive power, with adjusted R^2^ values ranging from 0.939 to 0.971. The regression equations illustrate the direction and magnitude of each variable’s influence on the number of working days lost due to sick leave.

The multivariate logistic regression models (Table 6, Table 7, Table 8 and Table 9) confirmed the role of demographic and occupational variables as significant predictors of MSD-related absenteeism. Female staff, older workers (≥46 years), and nurses consistently exhibited higher odds of sickness absence, particularly for chronic MSDs. The diagnosis month and pandemic period also significantly influenced absenteeism trends. These findings were consolidated into a linear regression summary (Table 10), which quantified the contribution of each factor to the number of working days lost, with adjusted R^2^ values exceeding 0.93 across models, highlighting strong predictive reliability.

Although the analysis considered all age groups, only the ≥46 years category displayed a statistically significant (or near-significant) association with the outcome in the final adjusted model. Consequently, the results for this age group were explicitly reported in the table. Age was modeled as a binary variable (≥46 vs. <46) based on initial stratification, and younger age groups were included in the analysis but did not show statistically significant effects.

## 4. Discussion

This study highlights a significant increase in sickness absence due to musculoskeletal disorders (MSDs) among pediatric healthcare workers in the post-pandemic period. The data obtained identified several key risk factors associated with increased MSD-related absenteeism among pediatric HCWs, namely female gender, age ≥46 years, the nursing profession, and specific MSD diagnoses such as arthropathies and dorsopathies. Our findings are congruent with published data indicating a high prevalence of absenteeism due to MSDs among healthcare workers [6,26,35]. We also observed a change in the seasonality of absenteeism. These findings demonstrate the need for targeted occupational health interventions to reduce absenteeism and improve workforce sustainability.

### 4.1. Gender-Specific Trends

The results presented in Table 5 allow for a correlation of the observed trends with demographic, occupational, and diagnostic factors, providing insight into the reasons for the variation in sickness absence. In the pre-pandemic period, male HCWs took slightly longer sick leave on average than female HCWs (approximately 9.6 vs. 8.9 days), which may reflect higher exposure to physically demanding roles such as stretcher-bearers and maintenance staff, where trauma and osteoarticular conditions are more common [36]. During the pandemic, men demonstrated a more significant decline in sick days. This reduction may be attributed to fewer workplace accidents and lower exposure to respiratory infections due to protective protocols. In contrast, the decline in sick leave among female staff was more modest, likely due to their overrepresentation in roles such as nursing, where physical and psychological strain remained elevated [37]. Additionally, increased family responsibilities during the pandemic, including school closures and caregiving, may have influenced their patterns of leave [38,39]. Over 80% of MSD-related sick leave was taken by female HCWs (Table 1). Contributing factors include repetitive movements, prolonged standing, and awkward postures. These trends align with the prior literature documenting the gendered burden of healthcare roles [40,41,42,43]. Females display greater lumbar lordosis and lower spinal load tolerance than males, potentially due to their increased risk of MSDs during lifting tasks [44]. Broader influences, such as occupational division, biologically unadapted work design, and the additional load of unpaid domestic work, collectively intensify the female HCWs’ MSD risks [45,46,47,48].

Our data reinforce these patterns and highlight the need for gender-sensitive occupational health strategies. The seasonal distribution of MSD-related absences also demonstrates gendered variation, with female-dominated roles being more affected by spring peaks (Figure 1). Logistic and linear regression models (Table 6 and Table 7) further confirm that MSDs—especially arthropathies and dorsopathies—are more prevalent among women. In contrast, absences due to trauma and fractures decreased among women during the pandemic, likely due to mobility restrictions and fewer occupational injuries (Table 9). Moreover, our logistic regression results (Table 6, Table 7, Table 8 and Table 9) show significantly higher odds of MSD-related absence among women, particularly during the pandemic, indicating an increased impact on women from changes in workflow and protective requirements.

### 4.2. Age-Related Vulnerability

Chronic MSDs were the cause of higher absenteeism among HCWs aged 46 and older, indicating an age-related susceptibility that requires specialized ergonomic and occupational support. Studies from various settings—including Europe, Asia, and the Americas—have revealed similar trends of increased MSD-related absenteeism, particularly among older HCWs and nurses [2,6,17]. Older workers (≥46 years) experienced a longer average duration of sick leave prior to the pandemic, which is expected due to the accumulation of health issues with age (especially chronic osteo-articular, cardiovascular, etc.) [49,50,51,52]. The relationship between age, health status, and response to the pandemic environment is evident: those most at risk (older individuals with health problems) gained the most from protection and reorganization measures [35,53], resulting in a reduction in sick leave.

### 4.3. Occupational Differences

Another important predictor was occupation. Nurses—who face constant physical overexertion from patient handling, extended standing, and repetitive tasks—accounted for more than half of all absences linked to MSDs. In our study, nearly half of the MSD-related sick leave was granted to nurses (Table 1), confirming prior findings that they are the occupational group with the highest risk of absence due to MSDs [35,54,55]. This is attributable to the nature of their work, which involves frequent patient handling, prolonged awkward postures, and physically demanding tasks [35,54]. Palladino et al. (2022) suggest that factors such as vaccination, variant characteristics, management practices, and improved working conditions contributed to reduced sickness absence among healthcare workers during the pandemic period [56]. Schug et al. (2022) similarly found that perceived support, adequate staffing, and reward systems were linked to fewer sick days among nurses [57].

Frontline care workers with high physical demands—such as nurses, stretcher-bearers, and care assistants—experienced the highest sick leave rates pre-pandemic [58], likely due to tasks involving lifting patients and handling equipment. The significant drop in absenteeism in this group during 2020–2021 suggests that hospital measures introduced during the pandemic were effective [35,56]. Potential contributing factors include reduced workloads (via deferral of non-urgent cases or staff rotation), stricter safety protocols, vaccination, and investments in protective measures (e.g., ergonomic training and improved PPE).

Absenteeism among physicians also decreased, likely influenced by heightened professional commitment during the crisis [59]. Many doctors may have avoided taking leave unless it was essential, driven by a sense of responsibility and improved access to medical care [35].

During the COVID-19 pandemic, nurses’ absenteeism showed no variation, highlighting their ongoing high exposure and workload even during this period. Nurses were consistently overworked [60], remained the backbone of care [61], and many became ill—either with COVID-19 or from pre-existing conditions worsened by exertion [62,63]. Despite the official end of the acute pandemic phase, nurses continue to bear significant physical and psychological burdens. Barros and Baylina (2024) point to the persistence of psychosocial risks such as emotional overload, job insecurity, and strained workplace relationships [64]. Anxiety and depression remain common due to ongoing emotional demands and insufficient mental health support [65]. Giorgi et al. (2020) emphasize that extended workloads, chronic fatigue, and ineffective recovery efforts have left many nurses emotionally depleted [66].

In the post-pandemic period, MSDs continue to affect nurses, worsened by ergonomic strain [67], shift-related fatigue [68], and psychosocial stressors [69]. These issues are compounded by delays in ergonomic interventions [70] and ongoing psychological stress [71], which highlight the intricate relationship among physical discomfort, workplace tension, and mental health challenges. As many authors have suggested, in the post-pandemic period, unresolved systemic issues and prolonged stress sustain dissatisfaction, burnout, and long-term health consequences among nurses [56,64,65,66,72]. Without meaningful leadership and institutional reforms [66], the balance between work demands and nurses’ health has remained precarious [73]. Although some causes of absence (e.g., seasonal infections) declined due to hygiene measures, others (e.g., fatigue, anxiety, and COVID-related illness) may have increased [74,75,76].

The experience of administrative staff illustrates how the working environment impacts health: office workers saw a significant drop in sick leave during the pandemic due to reduced physical contact, but rates returned to normal afterward, suggesting that traditional absenteeism returned with pre-pandemic routines [77].

The disproportionately high absenteeism among female nurses can be understood through the Job Demand–Control–Support framework, which emphasizes the role of high demands, low autonomy, and insufficient social support in occupational stress. Similarly, the Biopsychosocial Model is reflected in our discussion of how physical load, psychological stress, and organizational context jointly affect musculoskeletal health (Table 11).

### 4.4. Diagnostic-Specific Patterns

The duration of absence varies by type of illness (Table 4). Both calendar and working days were analyzed due to medical staff schedules, including non-working on-call days. Trends in both measures were similar: arthropathies and traumas resulted in longer absences than dorsopathies, aligning with the existing literature [6,32]. The univariate analysis showed that dorsopathies had the shortest mean sick leave—5.69 ± 3.43 working days and 7.38 ± 4.86 calendar days—while arthropathies had the longest—7.53 ± 4.41 working days and 10.14 ± 6.24 calendar days (*p* = 0.001) (Table 4).

The multivariate regression confirmed that the pandemic period significantly predicted increased MSD-related absenteeism due to both arthropathies and dorsopathies, particularly among female HCWs and those aged 46 and above (Table 6 and Table 7). These findings support the hypothesis that occupational factors (e.g., altered workflows, PPE use, and physical exhaustion) and indirect effects (e.g., delayed diagnosis, ergonomic changes, and mental fatigue) are associated with a heightened MSD burden post-pandemic. However, due to the observational nature of this study, we cannot infer a direct causal relationship.

The nature of illness-related absence offers insight into how HCWs were affected by the pandemic. Dorsopathies—primarily, spinal disorders and back pain—remain a leading cause of absence, often linked to physical strain from lifting and prolonged standing [81,82]. However, the average duration of dorsopathy-related leave declined significantly during the pandemic, suggesting ergonomic improvements or reduced overuse [83]. Possible explanations include greater availability of patient-handling equipment and decreased volumes of non-urgent procedures. Similarly, acute traumas such as injuries or fractures resulted in fewer absences post-pandemic compared to the previous period, possibly reflecting improved workplace safety and injury prevention efforts during a time when the availability of HCWs was critical. In contrast, chronic arthropathies (e.g., gonarthrosis and coxarthrosis) maintained steady absenteeism levels, implying that degenerative conditions required consistent recovery time regardless of pandemic-related changes. Other osteo-articular disorders—possibly tendinitis and connective tissue diseases—showed no significant variation, reinforcing that chronic MSDs remained a persistent cause of absence. Conversely, acute or workload-sensitive conditions (e.g., back pain and trauma) improved during the pandemic, likely due to workplace adaptations. These findings partially diverge from prior studies [26,84].

### 4.5. Temporal and Seasonal Patterns

Most studies have focused on general MSD prevalence and occupational risk factors, with limited attention to seasonal dynamics or comparisons to broader absenteeism trends [2,6,21,58,85]. These highlight a gap in the literature and an opportunity for future research to determine whether observed seasonal patterns are MSD-specific or reflect broader occupational health trends.

An analysis across pre-pandemic, pandemic, and post-pandemic periods revealed a U-shaped trend in MSD-related absenteeism: absences declined but then rose again in the post-pandemic period, often surpassing pre-pandemic levels (Table 1). This pattern suggests a dual association—initial relief from physical demands at the onset of the pandemic, followed by a resurgence of chronic musculoskeletal issues that may have been influenced by limited access to care, prolonged stress, and evolving workplace conditions (Table 5).

These findings align with international studies reporting increased MSD symptoms among healthcare workers during and after the pandemic [17,24,27]. For example, Zerbo Šporin et al. (2025) noted similar post-pandemic increases in absenteeism among Slovenian healthcare workers [17], while Efe et al. (2021) connected reduced physical activity and heightened stress during the pandemic to increased musculoskeletal pain and subsequent sick leave [27]. Furthermore, published data indicate that delayed medical care and pandemic-related changes in working conditions—such as PPE use and altered routines—exacerbated pre-existing symptoms and contributed to overload of the musculoskeletal system [25,86,87,88].

Notably, post-pandemic peaks in MSD-related absences shifted from autumn and winter (October–December) to spring and early summer (March, May, and June)—a trend previously underexplored in the literature. Seasonal variation also differed by diagnosis: spinal disorders peaked in late winter and summer, arthropathies in spring and summer, and trauma at the beginning and end of the year (Table 6, Table 7, Table 8 and Table 9). December, used as the reference month in the models, consistently showed the lowest absence rates, while the risk of absence was significantly higher in the identified peak months.

Age over 46 had a modest yet consistent effect on absenteeism due to osteo-articular disorders. Specifically, regarding arthropathies and dorsopathies, staff in this age group exhibited slightly higher absence rates (Table 6 and Table 7). In contrast, age had minimal impact on absences related to trauma or other acute conditions. The duration of sick leave also varied by diagnosis and reflected severity: dorsopathies and trauma were associated with longer absences (Table 8 and Table 9), while extended leave durations were not specifically linked to arthropathies.

### 4.6. Limitations of This Study

This study was conducted in a single pediatric medical unit, which limits the generalizability of the results to other medical units. It does not account for variations in the severity of illness, which may influence the duration of sickness absence. Additionally, pre-existing health conditions (e.g., obesity) and psychosocial factors (psychological stress and burnout) that may contribute to sickness absence were not analyzed. The analysis relied on existing records of sickness absence, which may omit some factors. Some health professionals likely experienced repeated sick leave episodes; however, these were analyzed as independent events. The lack of individual-level longitudinal tracking precludes the assessment of recurrent absenteeism within the same worker and may introduce clustering effects, potentially biasing statistical significance. This represents a key limitation that future studies should address through longitudinal cohort designs. The anonymized data used comes from administrative records, which may not fully capture the factors influencing absenteeism. The transition from “pandemic” to “post-pandemic” was not abrupt. Pandemic waves still characterized the first half of 2022. As a limitation, the 2022 data are included in the post-pandemic period, although some may still be directly influenced by the pandemic. This may reduce the contrast between the pandemic and the post-pandemic.

While our study provides detailed insights into MSD-related absenteeism in a pediatric hospital setting, direct comparisons with national figures remain limited due to the lack of disaggregated, occupation-specific data in Romanian national statistics. Reports from Romanian public health institutions do not routinely publish sickness absence data categorized by diagnosis (e.g., MSDs) or professional group, which restricts our ability to benchmark our findings against national averages. However, findings from broader European datasets and studies in similar occupational contexts suggest that the trends, particularly the higher absenteeism rates among women, nurses, and older HCWs, are consistent with patterns reported in other healthcare environments. For instance, Zerbo Sporin et al. (2025) examined Slovenia’s national MSD absenteeism and discovered comparable post-pandemic rises and demographic risk variables [17]. Considering the shortage of comparative information at the national level in Romania, this study provides a regional context. It highlights the need for more detailed, openly accessible national data on absenteeism due to MSDs and occupational health. The association between heightened psychosocial stress and MSDs among HCWs has also been confirmed across international contexts, underscoring the need for integrated occupational health strategies that address both physical strain and mental well-being [64,65,66]. Together, this body of evidence supports the broader relevance of our findings beyond the Romanian healthcare system.

The regional children’s hospital where the study was conducted offers more than 30 medical and surgical specialties, a laboratory, an emergency department, and an outpatient clinic. This study benefits from a substantial number of cases analyzed (N = 3388 sick leaves) and a statistical analysis utilizing logistic and linear regression models. These aspects enhance the reliability of the results. However, future studies should include multicenter analyses and assess disease severity and psychosocial factors to improve predictive models and better understand the interaction between occupational stress and absenteeism due to MDS.

Finally, as an observational and retrospective analysis, our study identifies statistically significant associations between various demographic, occupational, and temporal variables and MSD-related absenteeism. However, causality cannot be inferred. Potential confounding factors (e.g., pre-existing conditions, coping strategies, or institutional variations in policy) were not fully controlled for and may influence the observed relationships.

### 4.7. Implications for Policy and Practice

Statistical models indicate that variables such as the pandemic period, month of diagnosis, age, and duration of sickness account for 94–97% of the variation in absence duration across diagnostic groups (Table 10). This suggests that contextual factors (e.g., pandemic and seasonality) and patient or illness characteristics significantly influence the incidence and duration of sickness absence. Absenteeism due to MSDs—particularly arthropathies, dorsopathies, and other osteoarticular conditions—was influenced by gender (with women being more affected); pandemic context (which increased chronic condition-related absences and reduced trauma-related ones); seasonal trends; and, to a lesser extent, age. Trauma and fractures followed a distinct trajectory, showing lower incidence during the pandemic but longer recovery periods when they did occur. These patterns summarize key factors associated with MSD-related sickness absence across the pre-, during-, and post-pandemic periods. While these associations are statistically significant, they do not establish causality.

In correlating these factors, our data demonstrate that high-risk healthcare workers (HCWs)—those who are older, male, engaged in physically demanding roles, or have histories of dorsopathy or trauma—experienced notable improvements in sickness absence during the pandemic (Table 5). Thus, the interventions implemented during this period, whether organizational, preventive, or cultural, positively impacted vulnerable segments of the workforce. Conversely, groups that showed no improvement (e.g., younger staff, nurses, and those with chronic degenerative conditions) seem to have been less affected by these interventions, highlighting the need for more targeted support in those categories.

Published evidence supports the link between psychological distress—particularly anxiety and depression—and the rise in MSDs during the pandemic [65,66]. Psychosocial stressors such as high workload, emotional exhaustion, and insufficient workplace support significantly mediate this relationship [64]. These results highlight the importance of incorporating ergonomic and psychosocial metrics into occupational health programs. In the post-pandemic recovery phase, reducing the total burden of occupational sickness requires developing compassionate leadership, improving mental health assistance, and establishing psychologically safe work environments.

From an occupational health policy perspective, our results highlight the ongoing burden of MSDs and associated absenteeism among pediatric HCWs, particularly nurses. These facts necessitate proactive workforce strategies that emphasize ergonomic improvements, routine psychosocial risk assessments, and flexible staffing models to alleviate overwork and burnout [89]. Instead of relying on reactive measures, occupational health policies should incorporate regular physical and mental health screenings, tailored return-to-work programs, and interdisciplinary rehabilitation services [90,91]. At the system level, tackling chronic understaffing will require government investment in recruitment and retention, along with supportive leadership structures [92]. For Romania, this necessitates integrating mandatory health evaluations and job adaptation plans into occupational health law [93], as well as initiatives like recruitment incentives, bonuses, and work-life balance policies to address public sector staff shortages [94]. Leadership training should also prioritize psychological safety, communication, and burnout prevention [95]—all essential for maintaining a resilient, effective healthcare workforce.

Our analysis indicates a general trend toward reduced sickness absence duration among HCWs in regional pediatric hospitals during the pandemic and immediate post-pandemic period compared to pre-pandemic levels. This improvement was most notable among older male staff and those in physically demanding roles. Contributing factors likely included enhanced protection against infectious diseases (e.g., lower flu incidence) [96], improved ergonomics and injury prevention [97,98], and greater awareness of health risks among both workers and employers [99,100,101]. Hospital restructuring may also have alleviated some departments’ sick leave burden [102].

However, chronic degenerative conditions like arthropathies continue to result in substantial work incapacity, indicating a need for focused occupational health interventions [103,104]. These should include regular health check-ups (clinical and imaging assessments), early treatment of osteoarticular issues, physiotherapy, strengthening exercises, and modified job roles (e.g., task rotation or reduced joint loading). The lack of improvement among nurses suggests this group would particularly benefit from additional support to reduce stress and burnout [8], such as increased staffing, access to psychological services, and encouragement to use vacation leave. Researchers can statistically model predictors of sickness absence using demographic and clinical variables [17,55,105,106,107]. Predictive modeling offers hospital administrators a valuable tool for anticipating high-risk periods, enhancing resource planning, and supporting workforce and care continuity. Given the increased risk of MSD-related absence among pediatric staff, we recommend several preventive strategies: (i) regular screening and ergonomic counseling to reduce musculoskeletal strain; (ii) individualized recovery programs involving occupational health specialists; (iii) flexible work schedules to mitigate seasonal peaks; and (iv) predictive models to inform staffing decisions. These measures can reduce the economic impact of absenteeism, protect worker health, and preserve care quality. Further long-term research will be critical to clarifying causal pathways between risk factors and MSDs and refining evidence-based interventions. These findings offer actionable insights into how demographic and occupational factors influence MSD-related absence and how hospitals can proactively manage staffing capacity through targeted risk reduction, rehabilitation, and wellness programs.

## 5. Conclusions

This study highlights the increase in sickness absence due to MSDs in the post-pandemic period, featuring significant seasonal variations and a greater impact on women, nurses, and workers over the age of 46. These findings underscore the need for urgent occupational health interventions to prevent staff loss. Future research should investigate the severity of the illness and the effectiveness of prevention strategies. Healthcare institutions need to implement job-specific ergonomic counseling programs and predictive models to reduce musculoskeletal strain and maintain quality of care. Addressing these issues will contribute to a more resilient and sustainable healthcare system.

## Figures and Tables

**Table 1:** Descriptive epidemiological data on the causes of absenteeism at the regional pediatric hospital in Iasi, Romania.

	Arthropathies		Dorsopathies		Other MSD Diseases		Trauma/Fracture		Total		Chi Square Test
	No. of Cases	%	No. of Cases	%	No. of Cases	%	No. of Cases	%	No. of Cases	%	*p*
	217	6.40	2116	62.46	147	4.34	908	26.80	3388	100	
Period											
2017–2019	64	29.5	597	28.2	40	27.2	318	35.0	1019	30.1	
2020–2021	68	31.3	693	32.8	41	27.9	207	22.8	1009	29.8	0.001
2023–2023	85	39.2	826	39.0	66	44.9	383	42.2	1360	40.1	
Gender											
Male	17	7.9	269	12.8	10	6.8	150	16.6	446	13.2	
Female	199	92.1	1833	87.2	137	93.2	754	83.4	2923	86.8	0.001
Age											
<46 years	40	18.4	783	37.3	56	38.1	379	41.9	1258	37.3	
≥46 years	176	81.5	1319	62.7	91	61.9	525	58.1	2111	62.7	0.001
Occupation											
Physician	19	8.8	279	13.2	13	8.8	118	13.0	429	12.7	
Nurse	114	52.5	985	46.6	80	54.4	403	44.4	1582	46.7	0.025
Porter, carer	63	29.0	537	25.4	40	27.2	270	29.7	910	26.9	
Registrar	8	3.7	118	5.6	6	4.1	52	5.7	184	5.4	
Auxiliary staff	1	0.5	39	1.8	3	2.0	15	1.7	58	1.7	
Administrative staff	10	4.6	112	5.3	3	2.0	34	3.7	159	4.7	
Other HCWs with higher ed. qualification	-	*-*	20	1.0	2	1.4	11	1.2	33	1.0	

**Table 2:** Descriptive statistical indicators of the number of total calendar days of sick leave.

N		3388
Mean		8.25
Median		6
Standard Deviation		5.86
Variance		34.32
Skewness Test		1.335
Skewness Standard Error		0.042
Minimum		1
Maximum		31
Percentiles	25th	4
	50th	6
	75th	12

**Table 3:** Descriptive statistical indicators of the number of total working days of sick leave.

N		3388
Mean		6.14
Median		5
Standard Deviation		4.03
Variance		16.22
Skewness Test		1.160
Skewness Standard Error		0.042
Minimum		0
Maximum		23
Percentile	25th	3
	50th	5
	75th	9

**Table 4:** Univariate analysis: descriptive clinical data by causes of absenteeism.

Clinical Characteristics	Arthropathies (n = 217)	Dorsopathies (n = 2116)	Other MSDs (n = 147)	Trauma/Fracture (n = 908)	Total Lot (n = 3388)	Chi-Square Test *p*
Calendar days						
Mean ± SD	10.14 ± 6.24	7.38 ± 4.86	8.25 ± 5.09	9.83 ± 7.38	8.25 ± 5.86	0.001
Min–Max	1–31	1–31	1–23	1–31	1–31	
Working days						
Mean ± SD	7.53 ± 4.41	5.69 ± 3.43	6.16 ± 3.61	6.87 ± 5.00	6.14 ± 4.03	0.001
Min–Max	0–21	0–22	0–17	0–23	0–23	

**Table 5:** Average number of sick leave days (mean ± SD) taken by HCWs, categorized by gender, age, MSDs, and occupation, during the pre-pandemic (2017–2019), pandemic (2020–2021), and post-pandemic (2022–2023) periods. Changes over time were assessed using ANOVA; *p*-values <0.05 indicate statistically significant differences among the three time periods.

Characteristics of the Analyzed Group of HCWs	Mean Value (± Standard Deviation) of Calendar Days of Sick Leave In			Analysis of Variance (ANOVA) Comparing the Pre-Pandemic, Pandemic, and Post-Pandemic Periods
Gender	Pre-pandemic period	Pandemic period	Post-pandemic period	*p*-value (ANOVA)
Male	9.56 ± 7.19	7.41 ± 5.87	6.96 ± 5.31	0.001
Female	8.92 ± 6.15	8.27 ± 5.62	7.87 ± 5.66	0.001
Age	Pre-pandemic period	Pandemic period	Post-pandemic period	*p*-value (ANOVA)
<46 y	8.56 ± 6.50	7.97 ± 5.91	7.62 ± 5.64	0.069
≥46 y	9.42 ± 6.14	8.27 ± 5.41	7.83 ± 5.62	0.001
Diseases	Pre-pandemic period	Pandemic period	Post-pandemic period	*p*-value (ANOVA)
Arthropathies	10.84 ± 6.79	10.26 ± 6.02	9.62 ± 6.00	0.432
Dorsopathies	7.95 ± 4.95	7.35 ± 4.64	6.86 ± 4.93	0.001
Other MSDs	8.35 ± 5.01	7.66 ± 4.76	8.56 ± 5.37	0.668
Trauma/fracture	10.72 ± 8.01	9.62 ± 7.74	9.21 ± 6.54	0.023
Occupation	Pre-pandemic period	Pandemic period	Post-pandemic period	*p*-value (ANOVA)
Physician	8.55 ± 7.05	6.15 ± 5.99	5.20 ± 4.10	0.001
Medical assistant	8.81 ± 6.32	8.46 ± 5.51	8.35 ± 5.97	0.456
Carer, porter	10.20 ± 6.68	8.96 ± 5.85	7.84 ± 5.53	0.001
Medical registrar	7.63 ± 4.95	7.94 ± 4.62	8.36 ± 5.02	0.615
Auxiliary staff	6.71 ± 4.15	7.82 ± 3.34	8.09 ± 5.14	0.549
Administrative staff	8.76 ± 4.94	6.28 ± 3.89	8.73 ± 4.64	0.009
Other staff with higher education	5.11 ± 3.25	5.82 ± 4.31	5.09 ± 3.51	0.401

**Table 6:** Logistic regression models predicting absenteeism due to arthropathy, adjusted for clinical data.

Logistic Regression Models Tailored Model	Independent Variables	OR (95% CI)	*p*-Value
Arthropathy Gender (F)			
Time frame	Pre-pandemic (1)	8.000 (4.274–6.656)	0.001
	Pandemic (2)	21.667 (6.810–68.936)	0.001
Month of diagnosis	January	5.765 (0.618–53.669)	0.124
	March	7.132 (1.993–25.514)	0.003
	April	2.308 (0.561–9.490)	0.246
	May	14.804 (1.935–113.25)	0.009
	June	2.498 (0.939–6.643)	0.067
	July	10.381 (2.296–46.934)	0.002
	November	11.541 (1.408–94.624)	0.023
Age	≥46 years	1.857 (1.039–3.526)	0.003
Number of calendar days of sick leave		1.043 (0.931–1.168)	0.246
Number of working days lost due to sick leave		1.043 (0.965–1.128)	0.284

**Table 7:** Logistic regression models for predictors of dorsopathy, adjusted for clinical data.

Logistic Regression Models Tailored Model	Independent Variables	OR (95% CI)	*p*-Value
Dorsopathy Gender (F)			
Time frame	Pre-pandemic (1)	5.333 (3.646–6.656)	0.001
	Pandemic (2)	7.378 (5.858–9.292)	0.001
Month of diagnosis	January	4.572 (2.873–7.277)	0.001
	February	13.539 (6.832–26.831)	0.001
	March	8.129 (4.876–13.554)	0.001
	April	7.210 (4.170–12.465)	0.001
	May	4.290 (2.838–6.485)	0.001
	June	3.847 (2.526–5.941)	0.001
	July	9.271 (5.272–16.306)	0.001
	August	3.559 (2.412–5.250)	0.001
	September	5.857 (3.805–9.017)	0.001
	October	6.779 (4.329–10.615)	0.001
	November	7.416 (4.329–10.615)	0.001
Age	≥46 years	1.302 (1.000–1.903)	0.006
Number of calendar days of sick leave		1.170 (1.122–1.219)	0.001
Number of working days lost due to sick leave		0.917 (0.827–1.016)	0.098

**Table 8:** Logistic regression models predicting absenteeism due to other diseases of the osteoarticular system and connective tissue, adjusted for clinical data.

Logistic Regression Models Tailored Model	Independent Variables	OR (95% CI)	*p*-Value
Other musculoskeletal disorders Gender (F)			
Period	Pre-pandemic (1)	7.873 (5.358–69.893)	0.001
Month of diagnosis	January	6.289 (1.765–51.697)	0.087
	March	8.124 (1.010–65.317)	0.049
	September	8.836 (1.116–69.976)	0.039
	October	3.473 (0.717–16.830)	0.122
	November	2.089 (0.390–11.185)	0.390
Age	≥46 years	1.058 (0.899–1.090)	0.058
Number of calendar days of sick leave		1.410 (0.034–5.470)	0.619
Number of working days lost due to sick leave		0.784 (0.208–2.138)	0.635

**Table 9:** Logistic regression models predicting absenteeism due to acute trauma/fracture pathology adjusted for clinical data.

Logistic Regression Models Tailored Model	Independent Variables	OR (95% CI)	*p*-Value
Trauma/injuries/fractures Gender (F)			
Period	Pre-pandemic (1)	4.925 (3.665–6.616)	0.001
	Pandemic (2)	2.764 (2.030–3.762)	0.001
Month of diagnosis	January	5.356 (2.882–8.448)	0.001
	March	6.257 (3.000–13.051)	0.001
	April	5.276 (2.612–10.655)	0.001
	May	4.506 (2.343–8.664)	0.001
	June	3.367 (2.013–5.631)	0.001
	July	5.282 (2.931–9.520)	0.001
	August	4.802 (2.594–8.891)	0.001
	September	2.685 (1.622–4.445)	0.001
	October	2.810 (1.708–4.622)	0.001
	November	6.252 (3.423–11.418)	0.001
Age	≥46 years	1.052 (0.653–1.740)	0.049
Number of calendar days of sick leave		1.520 (1.018–1.987)	0.003
Number of working days lost due to sick leave		1.049 (0.915–1.204)	0.491

**Table 10:** Summary of linear regression models predicting working days of sick leave by diagnosis type.

Diagnosis	Adjusted R^2^	Std. Error	Regression Equation	*p*-Value
Arthropathy	0.964	1.176	Y = 1.21 − 0.45 (Period) − 0.01 (MonthDg) − 0.03 (Age) + 1.39 (DaysC)	0.001
Dorsopathy	0.939	1.2	Y = −0.177 − 0.086 (Period) − 0.009 (MonthDg) − 0.001 (Age) + 1.374 (DaysC)	0.001
Other MSDs	0.95	1.135	Y = 0.694 + 0.022 (Period) − 0.046 (MonthDg) − 0.014 (Age) + 1.371 (DaysC)	0.001
Trauma/Fracture	0.971	1.265	Y = −0.678 − 0.043 (Period) + 0.007 (MonthDg) + 0.012 (Age) + 1.452 (DaysC)	0.001

**Table 11:** Theoretical models that explain work-related absenteeism in pediatric healthcare workers during the COVID-19 pandemic.

Model	Component	Real-World Application in This Study
Job Demand–Control–Support (JDCS) Model [78,79]	Job Demands	Pediatric nurses face frequent lifting of patients, prolonged standing, emotionally intense care, and increased workload during the pandemic.
	Job Control	Limited autonomy over shift schedules, task allocation, and pacing of work.
	Social Support	Reduced peer and managerial support during COVID-19 increased isolation and stress.
	Implication	High demands, low control, and low support collectively raise occupational stress and risk of MSD-related absenteeism.
Biopsychosocial Model [80]	Biological Factors	Age-related vulnerability (>46 years), repetitive musculoskeletal strain, and diagnosis-specific risks such as dorsopathies.
	Psychological Factors	Increased emotional burden, anxiety, and burnout, especially during the pandemic and in high-responsibility care roles.
	Social Factors	Gender-based role expectations, work–home imbalance, and institutional ergonomics (e.g., poor lifting support).
	Implication	Absenteeism reflects the cumulative effect of interacting physical, psychological, and social stressors.

## Data Availability

All data from the first author and the corresponding author are available.

## Abbreviations

The following abbreviation is used in this manuscript:

%	Percentage
ANOVA	Analysis of Variance
CI	Confidence Interval
COVID-19	Coronavirus Disease 2019
DaysC	Number of Calendar Days of Sick Leave
DaysL	Number of Working Days of Sick Leave
F	Female
HCWs	Healthcare Workers
IBM	International Business Machines
ICD-10	International Statistical Classification of Diseases and Related Health Problems 10th Revision
IQR	Interquartile Range
JDCS Model	Job Demand–Control–Support Model
LR	Likelihood Ratio
Month Dg.	The Month of Disease Diagnosis
MSDs	Musculoskeletal Disorders
N	Number of Observations
OR	Odds Ratio
Period	The Pre-Pandemic (2017–2019), Pandemic (2020–2021), and Post-Pandemic (2022–2023) Periods
*p*-value	Probability Value (in statistical significance testing)
R^2^	Coefficient of Determination
SD	Standard Deviation
SE	Standard Error
SPSS	Statistical Package for the Social Sciences
Y	Years
